# Implementation of a national electronic health information system in Gabon: a survey of healthcare providers’ perceptions

**DOI:** 10.1186/s12911-020-01213-y

**Published:** 2020-08-24

**Authors:** Cheick Oumar Bagayoko, Jack Tchuente, Diakaridia Traoré, Gaetan Moukoumbi Lipenguet, Raymond Ondzigue Mbenga, Aimé Patrice Koumamba, Myriam Corille Ondjani, Olive Lea Ndjeli, Marie-Pierre Gagnon

**Affiliations:** 1 Centre d’Innovation et de Santé Digitale, DigiSanté-Mali, USTTB, Bamako, Mali; 2 Centre d’Expertise et de Recherche en Télémédecine et E-Santé, CERTES, Bamako, Mali; 3 Research Center in Primary Care and Social Services, Quebec, Canada; 4 Université de Bordeaux, Bordeaux, France; 5 Ministère en charge de la santé, Projet eGabon-SIS, Libreville, Gabon; 6 Université de Tours, Tours, France; 7 Faculty of Nursing Sciences, Université Laval, Quebec, Canada

**Keywords:** Health information system, Information system success, User acceptance, Healthcare providers, Gabon

## Abstract

**Background:**

The Health Information System (HIS) is a set of computerized tools for the collection, storage, management, and transmission of health data. The role of such tools in supporting the modernization of health systems, improving access to quality healthcare, and reducing costs in developing countries is unquestionable, but their implementation faces several challenges. In Gabon, a unique national electronic HIS has been launched. It will connect healthcare institutions and providers at all levels in the whole country.

**Objective:**

This study aims to explore and identify the factors influencing healthcare providers’ perceptions of the national electronic HIS in Gabon.

**Methods:**

A 44-item questionnaire based on the Information System Success Model (ISSM) was administered between February and April 2018 among 2600 healthcare providers across the country. The questions assessed the different aspects of the HIS that could influence its perceived impact on a 5-level Likert scale (from fully agree to totally disagree). The reliability and construct validity of the questionnaire were checked using Cronbach alpha and congeneric reliability coefficients. A logistic regression was used to identify the factors influencing healthcare providers’ perceptions of the system.

**Results:**

A total of 2327 questionnaires were completed (i.e. 89.5% response rate). The logistic regression identified five elements that significantly influenced perceived system impact: System Quality (Odds Ratio–OR = 1.70), Information Quality (OR = 1.69), Actual Use (OR = 1.41), Support Quality (OR = 1.37), and Useful Functions (OR = 1.14). The model explained 30% of the variance in providers’ perception that the national HIS leads to positive impacts.

**Discussion:**

The results show that healthcare providers’ perceptions regarding the positive impact of the national HIS in Gabon are influenced by their previous use of an HIS, the scope of their usage, and the quality of the system, information, and support provided to users. These results could inform the development of strategies to ensure adequate change of management and user experience for the implementation of the national HIS in Gabon, and eventually in other low resource environments.

## Background

Health information Systems (HIS) enable the collection, storage, management, and transmission of data related to activities of healthcare providers, health organizations, patients, and health consumers [1]. HIS include electronic health records (EHR), clinical and hospital management, epidemiological and public health information, clinical decision support, patient portals, and remote monitoring systems.

HIS are now implemented, at least partially, in several developed countries [2]. One of the major benefits of HIS is the capacity to generate timely information about patient and population health to support healthcare provision and management at all levels [1]. Improved coordination of care is also achieved through health information exchanges that allow information-sharing between all healthcare providers [2]. Moreover, HIS could help improve population health by processing large sets of epidemiological data in order to identify trends and adapt public health interventions [3].

Governments in developing countries are also investing in national HIS and expect that this will facilitate access to quality healthcare and help reduce costs [4, 5]. However, many previous HIS implementation initiatives in low-income countries have not been successful due to poor planning [6]. The first obstacle to the implementation of HIS in developing countries is the current state of health systems, which often lack basic infrastructure [7–9].

Therefore, in order to ensure that the conditions are in place for the successful operation of any given HIS in those countries, the behaviour of healthcare with respect to these technologies must be studied, along with the potential facilitators and threats to their adoption and integration [4]. Lack of cooperation on their part could seriously undermine its effectiveness [10, 11].

Studies focusing on the adoption of HIS in developing countries are scarce [4–9, 12, 13] and to the best of our knowledge, there was no such research in Gabon prior to our current project, stemming from a national initiative called e-Gabon put forward by the government in order to modernize the country’s infrastructure based on rapid advances in information technologies. The uniqueness of this initiative lies in the fact that it encompasses several sectors of service and notably healthcare. A national electronic health information system is thus being developed. The systemic goal is to allow the deployment of an integrated HIS that can be used to connect healthcare institutions and providers at all levels in the whole country. An external evaluation has been mandated in order to accompany the development of the project. One of the first tasks has been to assess the conditions for success of such large-scale projects, taking into account the perspectives of end-users, including health organizations, professionals, and patients. Healthcare providers’ perspective is particularly important and has to be taken into consideration since they are the ones who will have to integrate the new system into their practice, which involves significant changes [10, 11]. The success of HIS implementation goes beyond its technological dimension. HIS acceptance by healthcare professionals begins by taking into account their perspective and identifying and treating any potential source of resistance. For instance, Heeks [14] recommended reducing the gap between the design of information systems and the reality of clinical practice by applying “local improvisation,” which could translate as contextualization and implementation of the principle of reality. Heeks [14] identified four areas of activity that could help fill these gaps: 1) Identify the organizational realities, an approach requiring open communication and considering local reality as legitimate; 2) Improve local technical skills, including IT project skills; 3) Inform key players about the limitations of information systems and about the methods of evaluation and integration used; and 4) Analyze the “how” as well as the “what” for the implementation plan should be as well thought out as the technological solution itself.

In light of these recommendations, the present study seems crucial to better integrate the perspectives of healthcare providers and contextualize the technical and organizational issues of future HIS implementation.

### Objective

This study is part of the broader evaluation strategy of the Gabon national HIS initiative. The aim of this study was to identify the specific elements of direct healthcare providers’ perceptions of the impact of implementing such a national electronic health information system.

### Theoretical models of HIT acceptance

Technology acceptance is considered as an essential condition for its adoption and use [15]. In developed countries, acceptance of health information technology has been the focus of research since the 1990s. Over the last decade, rapid technological advances, notably with the expansion of mobile telecommunication networks, have stimulated the application of information and communication technologies (ICT) for health purposes in developing countries.

There is generally a variety of models used in evaluating the implementation of health ICT in developing countries. The Technology Acceptance Model (TAM), developed by Davis and collaborators [16, 17], and its many extensions, such as the Unified Theory of Acceptance and Use of Technology (UTAUT) [18], have been extensively applied for assessing technology acceptance. However, the adequacy of these models to study technology acceptance in the context of developing countries has been criticized or has shown mixed results [19]. For instance, the fact that technologies such as EHR are not widespread in the daily life of the population limits the relevance of some variables of the UTAUT [9].

According to many authors, those models should be adapted to the context of developing countries [4, 5, 19, 20]. Thus, researchers often decide to adapt these acceptance models either by adding new variables [5, 9, 19] or by deleting variables [8, 19]. The addition of variables is done to facilitate the consideration of cultural specificities or technical issues specific to developing countries [3, 8, 17]. For instance, an adaptation of the UTAUT has proved conclusive in the case of a study on HIS acceptance conducted in Cameroon [17]: the percentage of explained variance increased from 12 to 46% when the moderating effect of age was considered in the model.

Other models have also been used to study technology acceptance in developing countries. For instance, several authors used the DeLone and McLean Information System Success Model (ISSM) [21] to measure the success of implementing an HIS [22–26]. These studies generally support the use of this model when it comes to evaluating the success of implementation in a developing or resource-limited country. In Ethiopia, for instance, the study by Tilahun and Fritz [23] provided satisfactory results to support the relevance of this model in a low-resource system.

## Methods

### Study population and setting

The target population consisted of professionals in charge of direct patient care (physicians, nurses, midwifes, etc.) or people working in healthcare services (managers, administrators) employed in different health structures of the ten (10) health regions of Gabon. A convenience sample of twenty-six hundred (2600) participants was selected, using a proportional sampling reflecting the percentage of healthcare providers within each region. Trained research assistants were deployed within each region to recruit participants from selected healthcare organizations. Potential participants were presented the study objectives and, after obtaining their verbal consent, were invited to complete the survey.

### Theoretical model

The theoretical background of this study is inspired by the DeLone and McLean ISSM [20, 26]. This model recommends taking into account five variables to measure the success of the implementation of an information system: system quality (SQ), support quality (SupQual), information quality (IQ), actual use (AU), satisfaction, and net benefits (Impact).

**Fig. 1:**
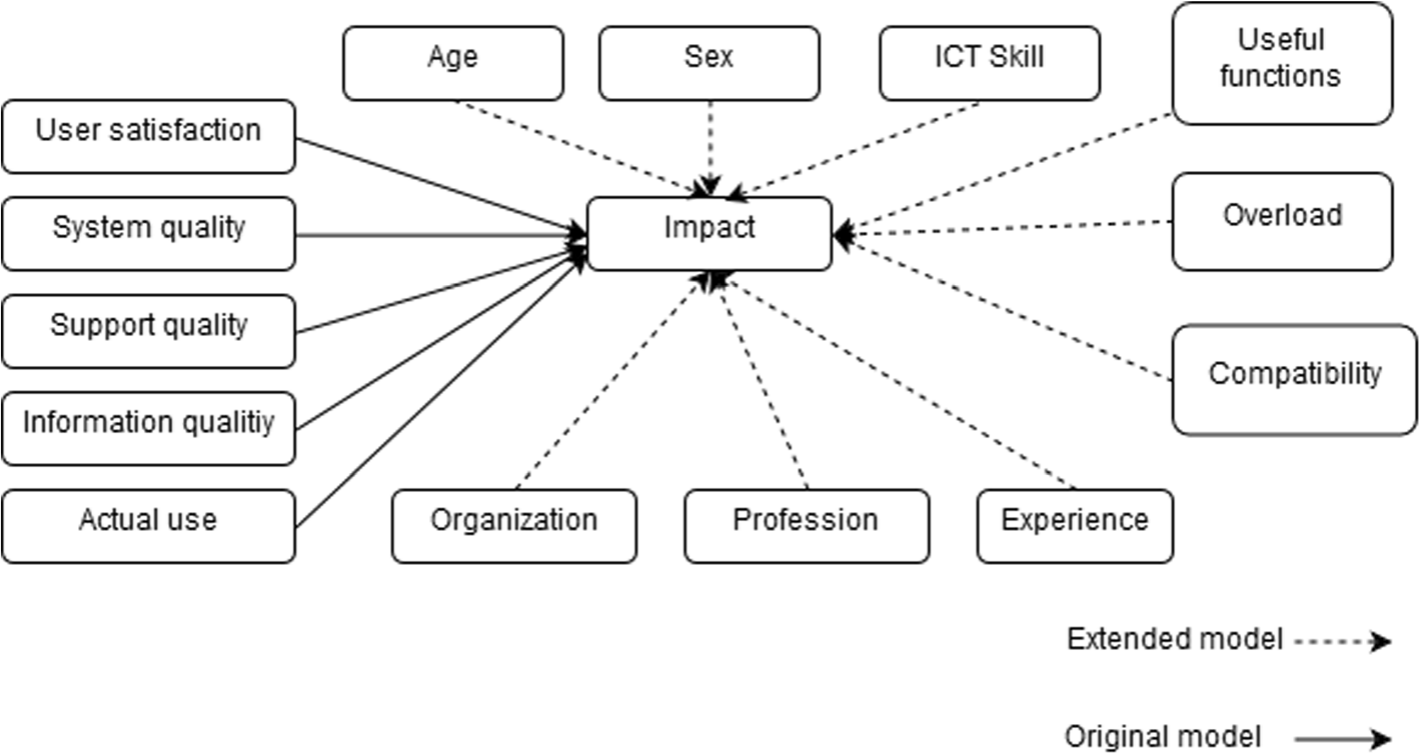
Theoretical model adapted from DeLone & McLean. The theoretical model is based on the DeLone and McLean Information System Success Model. This model proposes five variables to measure the success of an information system: system quality, service quality, information quality, actual use, satisfaction, and net benefits (impact)

In our case, we extended the ISSM by adding sociodemographic variables (age, sex, profession, experience, organization, and self-reported ICT skills) and psychosocial factors (useful functions, overload, and compatibility). Figure 1 presents the adapted theoretical model.

The survey questionnaire was developed based on previous studies using the ISSM and validated by members of the project team with expertise in medical informatics (COB), biostatistics (JT), and public health (MPG) to ensure clarity and cultural appropriateness of the wording (see Additional file 1). The questionnaire included fourteen (14) questions covering forty-four (44) items. Theoretical constructs were assessed by 5-point Likert scales and a “not applicable” option was available for participants who did not have previous experience of using an HIS.

### Data analysis

All responses were encoded into numerical values, and questionnaires with too many missing values were eliminated. First, we used Cronbach alpha and congeneric reliability (CR) coefficients to assess the internal consistency of each theoretical construct.

### Instrument reliability and validity

Table 1 shows that all constructs had satisfactory reliability. Cronbach alpha (Table 1) and CR (Additional file 2) were all above the minimally accepted value of 0.7 recommended to support the internal consistency of the constructs [27, 28]. All factor loadings were higher than 0.7 (Additional file 2). Fit indices were also within the recommend threshold, except for RMSEA which was slightly above [29] (Table 2).

**Table 1:** Internal consistency of theoretical constructs (Cronbach Alpha)

Construct	Cronbach alpha	No. Items	n
Satisfaction	0.85	4	1104
Support Quality (SupQual)	0.85	4	889
System Quality (SQ)	0.90	7	663
Impact	0.96	10	1036
Information Quality (IQ)	0.91	5	1009

**Table 2:** Fit Indices of the CFA

Model fit	Indicator value
RMSEA	0.071
CFI	0.925
IFI	0.925
TLI	0.918

Pearson correlations were performed between the different items as a first consistency check (Additional file 3). The coefficients were generally significant, high (> 0.4), and positive for items belonging to the same construct.

Confirmatory factor analysis (CFA) was used to assess construct validity. Observations with “not applicable” answers were eliminated for the validation of the theoretical model. However, they were kept for the computation of the construct if at least half of the items for the associated construct were valid responses.

### Normality and multicollinearity

Two assumptions have to be examined before performing a logistic regression. There should not be high multicollinearity between independent variables, and the dependent variable should not have a normal distribution. Correlations between variables are generally inferior to 0.3 (Additional file 4). However, some variables have a higher correlation score. For instance, SQ has a high correlation (> 0.5) with many variables such as Impact, IQ, or SupQual. As a high correlation may indicate multicollinearity, we performed a variance inflation factor (VIF) test. The VIFs obtained are all below five, which is the maximum value proposed by several authors [30–32] (Additional file 4). To test the normality, the Shapiro-Wilk test was performed on the dependent variable. The null hypothesis was rejected, which implied a non-normal distribution of Impact.

Due to the non-normality of the dependent variable, logistic regression was used in order to test the study hypotheses and identify the variables associated with perceived system impact (Impact) by healthcare providers. Ordinal variables with more than two modalities and Impact, the dependent variable, were dichotomized in order to perform the logistic regression. Age was dichotomized (“less than 40 years old” and “40 years old and above”). Work experience was also divided in two categories (“less than 10 years” and “10 years and more”). With respect to ICT skills, the average category was chosen as the cut-off point. For nominal variables (organization and profession), we also performed a dichotomization considering the mode of the series. Impact was dichotomized at the median while the other theoretical variables were considered as continuous variables.

An alpha level of 0.05 was set for testing the statistical significance for both correlation and regression analyses. All statistics were performed with SAS 9.4.

## Results

### Characteristics of participants

**Fig. 2:**
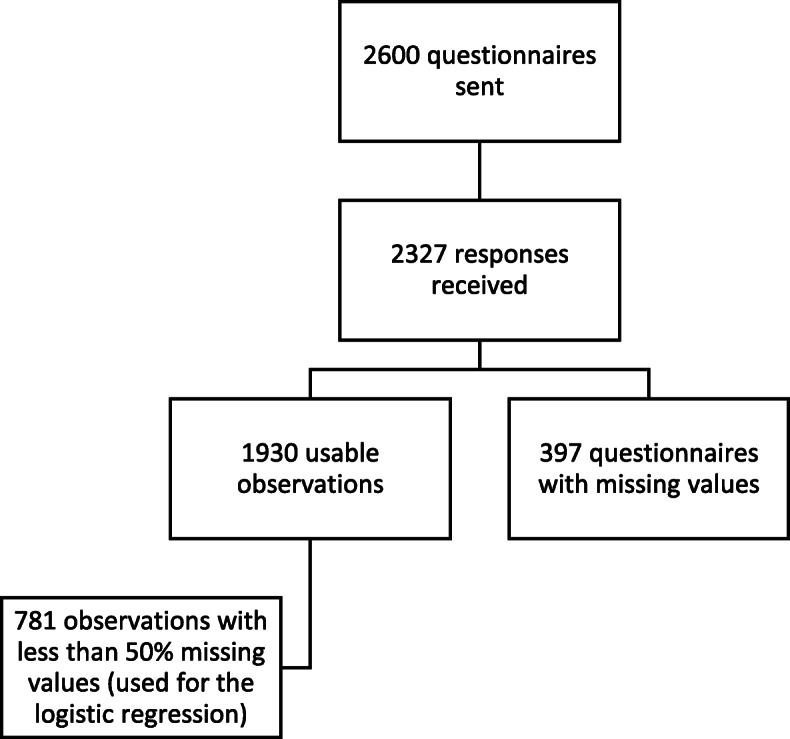
Study flow diagram. Of the 2600 potential participants, 2327 returned their questionnaires. There were 1930 usable questionnaires, and 781 questionnaires with complete observations were retained to test the theoretical model

Figure 2 presents the flow diagram of the study. A total of 2327 participants returned their questionnaires, for an overall response rate of 89.5%. There were 397 unusable questionnaires due to a high number of missing values, notably on socio-demographic questions. Of the 1930 questionnaires with usable observations, many included “not applicable” responses for some items, but they were kept for the reliability tests if at least 50% of the items had a valid response for a given construct. Finally, after eliminating questionnaires that had missing values and “not applicable” answers to one or more theoretical variables, we retained 781 questionnaires with complete observations in order to test the theoretical model (Fig. 2).

Table 3 presents the socio-demographic characteristics of respondents. Among the 1930 usable questionnaires, 1275 were completed by women (66.1%) and 655 by men (33.9%). The majority of participants (47.5%) were between 40 and 49 years old. Regarding ICT skills, a majority reported an average level (36.6%). However, more people (51.8%) reported having a low level (null or elementary) than a high level (advanced or expert) (11.6%). With respect to occupational characteristics, nurses constituted the majority at 55.5%, followed by professionals from a regional hospital (RH) at 29%. A majority of respondents had a long tenure in their profession, with 60.8% of them having been in their field for more than ten (10) years.

**Table 3:** Characteristics of participants

Variables	Categories	*n* (%)^a^
Age	Under 30	101 (5.2)
	30–39	583 (30.2)
	40–49	917 (47.5)
	50–59	301 (15.6)
	60 and above	28 (1.5)
Experience	0–5 years	323 (16.7)
	6–9 years	434 (22.5)
	10+	1173 (60.8)
ICT Skill	None	564 (29.2)
	Elementary	436 (22.6)
	Average	706 (36.6)
	Advanced	209 (10.8)
	Expert	15 (0.8)
Organization	Regional hospital	559 (29)
	Medical center	348 (18)
	Other structure	316 (16.4)
	University health center	303 (15.7)
	Health center	196 (10.2)
	Private structure	145 (7.5)
	Dispensary	63 (3.3)
Profession	Nurse	1072 (55.5)
	Other health profession	412 (21.3)
	Midwife	140 (7.3)
	General practitioner	115 (6)
	Administrator	105 (5.4)
	Specialist practitioner	86 (4.5)
Health Region	Estuaire (Libreville Owendo)	669 (34.7)
	Woleu-Ntem	296 (15.3)
	Ngounié	271 (14)
	Estuaire (Ouest)	182 (9.4)
	Haut Ogooué	146 (7.6)
	Ogooué Lolo	92 (4.8)
	Ogooué Ivindo	81 (4.2)
	Moyen Ogooué	75 (3.9)
	Ogooué Maritime	68 (3.5)
	Nyanga	50 (2.6)
Sex	Female	1275 (66.1)
	Male	655 (33.9)

^a^*N* = 1930

### Logistic regression

The logistic regression model was first tested with all independent variables (Table 4). Of these, four variables showed a significant odds ratio (*p* < 0.05): SQ, IQ, SupQual, and UF. All these variables had a positive coefficient, which means that the higher their values, the more likely the individual will evaluate the impact of the HIS as strong.

**Table 4:** Logistic Regression of the Full Model

Variables	Estimate	Estimate confidence interval		Odds ratio	*p*-values	Standardized estimate
Intercept	−5.68	−6.85	−4.51	0	0	0
Satisfaction	0.07	−0.11	0.26	1.08	0.44	0.05
SupQual	0.29	0.06	0.53	1.34	0.02	0.16
SQ	0.51	0.25	0.77	1.66	0	0.28
IQ	0.51	0.3	0.72	1.67	0	0.3
AU	0.19	−0.39	0.78	1.21	0.52	0.05
Compatibility	0.18	−0.35	0.71	1.2	0.51	0.05
Overload	0.14	−0.3	0.59	1.15	0.53	0.03
UF	0.12	0.03	0.21	1.13	0.01	0.12
Age (0–39)	−0.14	−0.53	0.24	0.87	0.47	−0.04
Sex (Male)	−0.08	−0.42	0.26	0.92	0.64	−0.02
Experience (0–9)	−0.11	−0.5	0.29	0.9	0.6	−0.03
Low ICT skill	0.19	−0.17	0.54	1.2	0.3	0.05
Profession (Nurse)	−0.01	− 0.37	0.35	0.99	0.95	0
Organization (Regional Hospital)	0.22	−0.13	0.56	1.24	0.23	0.06

AUC: 0.77; Nagelkerke r-square: 0.31; *n* = 781

We performed a stepwise regression by testing a model in which only the significant variables were kept (Table 5). Consequently, one additional variable was retained in the model, namely AU. Respondents who have already used an HIS were about 1.5 times more likely to consider that HIS would have a positive impact.

**Table 5:** Stepwise Logistic Regression of the Final Model

	Estimate	Estimate confidence interval		Odds ratio	*P*-Values	Standardized estimate
Intercept	−5.7	−6.67	−4.74		0	
SupQual	0.32	0.1	0.54	1.37	0.01	0.18
SQ	0.53	0.28	0.78	1.7	0	0.29
IQ	0.52	0.32	0.73	1.69	0	0.3
AU	0.34	0.01	0.68	1.41	0.04	0.09
UF	0.13	0.04	0.22	1.14	0.01	0.13

AUC: 0.78; Nagelkerke R-squared: 0.3; *n* = 781

**Fig. 3:**
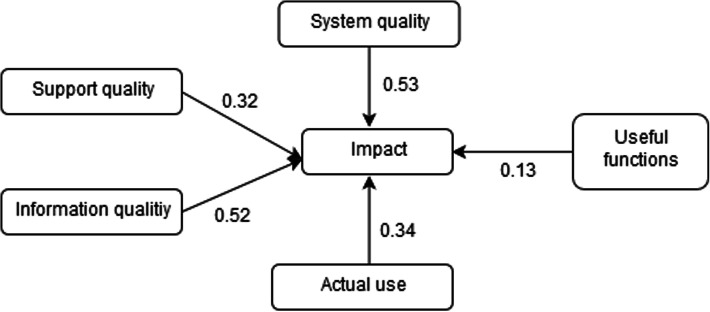
Final theoretical model with estimates. The final theoretical model explains 30% of the variance in providers’ perception of the positive impact resulting from the use of the HIS. Five variables of the adapted model are statistically significant, namely Support Quality, Information Quality, System Quality, Actual Use, and Useful Functions

The final model was able to explain 30% of the variance in the dependent variable. Using the leave-one-out cross validation method, we calculated the area under the curve (AUC). The model was able to correctly predict 78% of the cases (AUC = 0.78) (Fig 3).

The final model is represented by the following equation:

$$ \mathrm{logit}\left(\mathrm{p}\right)=-5.7+0.32\mathrm{SupQual}+0.53\mathrm{SQ}+0.52\mathrm{IQ}+0.34\mathrm{AU}+0.13\mathrm{UF} $$

Where p is the probability that Impact = 1.

## Discussion

This study aimed to identify the individual determinants of healthcare providers’ perceptions regarding the impact of a national electronic HIS in Gabon. We used a model adapted from the Information System Success Model [21, 33] that showed very good measurement properties and acceptable predictive power. In fact, a total of 30% of the variance in providers’ perceptions of the positive impact resulting from the use of the HIS was explained by five variables of our adapted model, namely Support Quality, Information Quality, System Quality, Actual Use, and Useful Functions. This latter variable was added to the initial ISSM in order to consider not only the actual use of an HIS but also the extent of its use by asking participants to indicate which specific functions of the HIS they use. The more functions they used, the higher their score. This variable could be related to the concept of ‘meaningful use’ of an information system by healthcare providers, which implies they optimally use the core functionalities of an information system [34].

There are very limited data from similar studies in low income countries. A study in Ethiopia [23] applied an adaptation of the ISSM and found that system quality, information quality, and service quality had a significant influence on EMR use and user satisfaction. Both actual use and user satisfaction were significantly associated with the perceived benefit of EMR. Surprisingly, our results do not support the influence of user satisfaction on perceived benefits since this construct is not significantly associated with Impact in the final model. A plausible explanation could be that the ISSM variables are seen as interdependent, meaning that other constructs, such as useful functions and actual use, might capture the influence of user satisfaction on perceived benefits [35].

A recent study conducted in Tanzania has tested a model that combines constructs from the ISSM and the TAM to understand user satisfaction and use of an EMR system [22]. The final model suggests that attitude and system quality are the only significant predictors of user satisfaction and system use.

Another study from Tanzania applied a modified ISSM to identify the factors influencing user satisfaction with an electronic logistic management information system [25]. The model includes perceived usefulness, a variable from the TAM, as well as facilitating conditions from the UTAUT. These two variables, together with system quality, information quality, and system support contributed significantly to the model, explaining 59.1% of the variance in user satisfaction.

Our adapted model allowed us to verify whether variables that are external to the ISSM could influence healthcare providers’ perceptions of the HIS. However, none of the added variables except useful functions significantly contributed to the model.

Some authors have suggested that inadequate computer literacy could hinder the success of EMR implementation in low income countries [36, 37], but this effect has not been rigorously tested [23]. In our study, we used a single self-reported measurement of computer skills but found no significant influence of this variable on healthcare providers’ perceptions of the HIS. It has been shown that self-reported computer literacy is not reliable because people tend to overestimate their skills [38]. However, the level of self-reported computer skills was generally low in our sample, making it unlikely that an overestimation would have biased the results.

### Implications for research and practice

The results from this study show that it is possible to adapt the ISSM model to a low-income setting, but the limited performance of the model in explaining healthcare providers’ perceptions calls for further research in order to better grasp the factors influencing HIS adoption in this context.

Based on our results and those of previous studies in similar contexts, it would be useful to include additional factors when assessing the conditions for successful implementation of health information systems in low income countries, notably the availability of adequate infrastructure, funding, and incentives [39].

Our results could inform the design of strategies to support the implementation of the national electronic HIS in Gabon and in other similar contexts, notably, the importance of ensuring the quality of the system and the information it contains. Ensuring that users are appropriately trained and supported is another key element for successful implementation. We also recommend that several useful functions be integrated within the HIS, helping users gain positive experiences and confidence in the usefulness of the system.

### Strengths and limitations

This study is one of the few focusing on the acceptance of a large-scale HIS in a low-income country and also one of the few that considers the perceptions of various groups of end users, including nurses, physicians, midwives, and health administrators. However, other important user groups working in Gabon’s healthcare structures, such as information technology staff and radiology, laboratory, and pharmacy technicians, were not included in the survey since the aim was to investigate the perceptions of direct healthcare providers. It would be interesting to survey these other groups given that their work will be directly impacted by the introduction of the national electronic HIS in Gabon.

Although we used an adaptation of a well-known theoretical model, the DeLone and McLean ISSM [21], the percentage of variance explained by this model is modest. Other models, such as the TAM and the UTAUT, are often applied to study user acceptance of HIS. However, some authors have questioned the appropriateness of such models in the context of low income countries [19]. In future studies, it could be interesting to compare different models in order to assess their applicability to the context of resource limited nations.

Our survey instrument showed very good psychometric properties, and we are confident that it could be adapted and used in other similar settings. Our theoretical model adapted from the ISSM is also promising but still needs improvement. Even though the scores obtained for the VIFs were relatively low, we cannot exclude the possibility of multicollinearity. Furthermore, the interdependence between the ISSM variables, particularly the mutual influence between use and user satisfaction, has been acknowledged previously and could constitute a limitation of this model [35, 40].

## Conclusion

The implementation of a national electronic HIS in Gabon represents a unique opportunity to modernize the health system and improve the management of healthcare and services to the population. The perceptions of healthcare providers are essential and have to be taken into consideration in order to ensure their acceptance and use of this system in a low resource setting. Using an adaptation of the Information System Success Model, this study found that information quality, system quality, support quality, actual use, and useful functions influenced the perception of positive impacts of the HIS by healthcare providers. Thus, to ensure the success of the implementation of the national HIS in Gabon, it is essential to involve healthcare providers in the design of this system and make sure that it can be incorporated in their clinical practice. Such cooperation is needed to ensure the quality of the system in terms of usability, quality of information, and end-user support. Potential users should also be trained in the various functionalities of the HIS so they can see and evaluate its benefits firsthand and increase the likelihood of its successful use.

## Supplementary information

**Additional file 1.** Survey questionnaire.

**Additional file 2.** Pearson correlations between items.

**Additional file 3.** Construct reliability of the model.

**Additional file 4.** Pearson correlations between constructs.

## Data Availability

All data are available from the corresponding author (COB).

## Abbreviations

AU: Actual use
CFA: Confirmatory Factor Analysis
CR: Congeneric Reliability
EMR: Electronic Health Record
EMR: Electronic Medical Record
HIS: Health Information System
ICT: Information and Communication Technology
IQ: Information Quality
SupQual: Support quality
SQ: System quality
UF: Useful functions
