# Supplementary material for: Implementation of a national electronic health information system in Gabon: a survey of healthcare providers’ perceptions
Source: BMC Med Inform Decis Mak. 2020 Aug 24;20:202. doi: 10.1186/s12911-020-01213-y (PMC7444076; doi:10.1186/s12911-020-01213-y)
Supplement: Supplementary file 1 — Additional file 1. Survey questionnaire. [file 12911_2020_1213_MOESM1_ESM.doc]

**Questionnaire : Satisfaction des professionnels de santé (*baseline*) sur le Système d’Information de Santé (SIS) et la e-santé**

**ID : Région: Dépmt: Ville: Structure :**

|  |
| --- |
| **DÉFINITION DES CONCEPTS UTILISÉS :** |
|  |
| ***Système d'Information de Santé :*** C'est l'ensemble des informations liées à tous les aspects de la santé d'un pays. Il a 2 composants principaux : le Système d'Information Sanitaire, du Système d'Information Hospitalier. De plus en plus, il a un troisième composante qu'est la e-santé.  ***Système d'Information Sanitaire***: Un système d'Information Sanitaire peut être défini comme un ensemble constitué d'acteurs, de ressources, d'outils et de méthodes qui interagissent à différentes étapes du processus de production de l'information sanitaire à savoir : la collecte des données, l'agrégation, le stockage, le partage et l'analyse des données. Il concerne particulièrement les informations sur tous les indicateurs de santé. Il a pour but de : i) de connaître la situation sanitaire et de fournir les éléments pour orienter la politique sanitaire; ii) de donner les éléments d’évaluation des actions de santé publique développées par les pouvoirs publics.  ***Système d'Information Hospitalier*** : Système destiné à traiter l'ensemble des informations médicales et administratives d'une structure de soins y compris la recherche et l'enseignement. Il a 3 fonctions principales : i) une fonction opérationnelle qui traite les informations mises en œuvre dans les processus de prise en charge des patients à l’hôpital ; ii) une fonction de pilotage de l’hôpital (décisionnelle) ; iii) une fonction de « *reporting* » vers le système d’information de santé et les différentes tutelles.  Le point de liaison de ces 2 composants est donc la gestion de l'information sanitaire. Lorsque le SIH est en place, il doit être en mesure d'alimenter le Système d'Information Sanitaire. C'est l'ensemble de ces informations qui constitueront le Système d'Information de Santé du pays. |
| ***e-Santé / m-santé*** : Selon l'Organisation Mondiale de la Santé (OMS), la e-santé se définit comme : " les services du numérique au service du bien-être de la personne". D'une façon générale il s'agit de l'utilisation des outils de production, de transmission, de gestion et de partage d'informations numérisées autant pour les pratiques médicales que médico-sociales. Elle inclut la télémédecine. Lorsque ces services sont accessibles via un appareil mobile ou une tablette on parle de ***m-santé***. |
|  |
| **Consentement :** |
| **Notez qu'en acceptant de répondre au questionnaire vous consentez à participer à l'étude. Soyez assurés que votre participation demeurera strictement confidentielle, de même que les réponses que vous fournirez.** |

**Q1.** En général, dans quelle mesure êtes-vous satisfait (e) du **Système d’Information de Santé (SIS) (et de la e-santé)** pour la réalisation de vos tâches et activités cliniques? La satisfaction concerne le fonctionnement et la facilité d’utilisation du système en soi, la qualité de l’information donnée par le système et la qualité des services de soutien qui vous sont offerts suite à son implémentation.

|  | **Entièrement satisfait(e)** | **Modérément satisfait(e)** |  | **Ni satisfait(e) ni insatisfait(e)** | **Peu satisfait(e)** | **Pas du tout satisfait(e)** | **Ne s’applique pas** |
| --- | --- | --- | --- | --- | --- | --- | --- |
| **Q1a**- Le fonctionnement du SIS | <INPUT TABINDEX=\ | <INPUT TABINDEX=\ |  | <INPUT TABINDEX=\ | <INPUT TABINDEX=\ | <INPUT TABINDEX=\ | 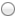 |
| **Q1b**- La facilité d’utilisation du SIS | <INPUT TABINDEX=\ | <INPUT TABINDEX=\ |  | <INPUT TABINDEX=\ | <INPUT TABINDEX=\ | <INPUT TABINDEX=\ | 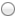 |
| **Q1c**- La qualité de l’information du SIS | 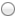 | 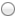 |  | 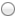 | 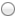 | 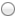 | 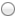 |
| **Q1d**- La qualité des services de soutien | <INPUT TABINDEX=\ | <INPUT TABINDEX=\ |  | <INPUT TABINDEX=\ | <INPUT TABINDEX=\ | <INPUT TABINDEX=\ | 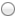 |

**Q2.** Veuillez indiquer dans quelle mesure vous êtes en accord avec chacun des énoncés ci-dessous.

|  | **Tout à fait en accord** | **Modérément en accord** |  | **Incertain (e)** | **Modérément en désaccord** | **Tout à fait en désaccord** | **Ne s’applique pas** |
| --- | --- | --- | --- | --- | --- | --- | --- |
| **Q2a**- Le processus d’implémentation du SIS était adéquat | <INPUT TABINDEX=\ | <INPUT TABINDEX=\ |  | <INPUT TABINDEX=\ | <INPUT TABINDEX=\ | <INPUT TABINDEX=\ | 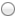 |
| **Q2b**- Le niveau de formation à l’utilisation du SIS est adéquat | <INPUT TABINDEX=\ | <INPUT TABINDEX=\ |  | <INPUT TABINDEX=\ | <INPUT TABINDEX=\ | <INPUT TABINDEX=\ | 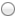 |
| **Q2c**- Le niveau de soutien offert à l’utilisation du SIS est adéquat | 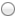 | 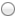 |  | 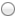 | 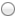 | 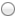 | 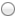 |
| **Q2d**- Il est facile d’accéder au SIS sur votre lieu de travail | <INPUT TABINDEX=\ | <INPUT TABINDEX=\ |  | <INPUT TABINDEX=\ | <INPUT TABINDEX=\ | <INPUT TABINDEX=\ | 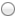 |

**Q3. D’après votre expérience jusqu’à présent, comment évaluez-vous la qualité du SIS par rapport aux caractéristiques spécifiques listées ci-dessous :**

|  | **Tout à fait en accord** | **Modérément en accord** | **Incertain (e)** | **Modérément en désaccord** | **Tout à fait en désaccord** | **Ne s’applique pas** |
| --- | --- | --- | --- | --- | --- | --- |
| **Q3a**- Le temps de réponse du SIS par rapport à une requête est acceptable | <INPUT TABINDEX=\ | <INPUT TABINDEX=\ | <INPUT TABINDEX=\ | <INPUT TABINDEX=\ | <INPUT TABINDEX=\ | 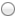 |
| **Q3b**- Le SIS est facile à utiliser | <INPUT TABINDEX=\ | <INPUT TABINDEX=\ | <INPUT TABINDEX=\ | <INPUT TABINDEX=\ | <INPUT TABINDEX=\ | 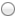 |
| **Q3c**- L’effort requis (ex. nombre de clics, défilement des items) pour compléter une action ou accéder à de l’information dans le SIS est acceptable | 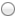 | 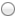 | 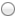 | 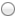 | 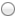 | 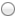 |
| **Q3d**- Le SIS permet d’assurer adéquatement la confidentialité et la sécurité des données personnelles des patients | 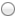 | 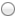 | 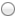 | 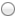 | 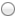 | 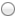 |
| **Q3e**- L’accès (mode de connexion) au SIS est acceptable | 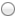 | 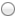 | 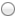 | 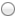 | 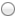 | 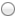 |
| **Q3f**- Le SIS a une performance fiable (ex. : le système est rarement en panne) | <INPUT TABINDEX=\ | <INPUT TABINDEX=\ | <INPUT TABINDEX=\ | <INPUT TABINDEX=\ | <INPUT TABINDEX=\ | 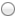 |
| **Q3g**- Le SIS permet de saisir toutes les informations que vous souhaitez | 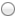 | 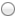 | 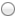 | 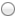 | 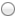 | 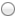 |

**Q4. Selon votre perception actuelle du Système d’Information de Santé (SIS), veuillez indiquer dans quelle mesure vous êtes en accord avec chacun des énoncés ci-dessous.**

|  | **Tout à fait en accord** | **Modérément en accord** | **Incertain (e)** | **Modérément en désaccord** | **Tout à fait en désaccord** | **Ne s’applique pas** |
| --- | --- | --- | --- | --- | --- | --- |
| **Q4a**- Le SIS accroît votre productivité | <INPUT TABINDEX=\ | <INPUT TABINDEX=\ | <INPUT TABINDEX=\ | <INPUT TABINDEX=\ | <INPUT TABINDEX=\ | 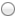 |
| **Q4b**- Le SIS facilite l’échange d’informations entre les différents utilisateurs du système (cliniciens et gestionnaires) | <INPUT TABINDEX=\ | <INPUT TABINDEX=\ | <INPUT TABINDEX=\ | <INPUT TABINDEX=\ | <INPUT TABINDEX=\ | 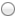 |
| **Q4c**- Le SIS améliore la gestion des soins et services de santé en temps réel | 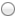 | 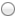 | 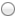 | 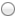 | 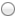 | 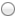 |
| **Q4d**- Le SIS améliore la gestion de l’information sur les patients en temps réel | 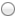 | 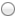 | 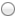 | 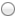 | 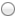 | 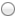 |
| **Q4e**- Le SIS permet d’accéder facilement à l’information souhaitée | <INPUT TABINDEX=\ | <INPUT TABINDEX=\ | <INPUT TABINDEX=\ | <INPUT TABINDEX=\ | <INPUT TABINDEX=\ | 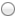 |
| **Q4f**- Le SIS permet de visualiser l’information relative à un patient | <INPUT TABINDEX=\ | <INPUT TABINDEX=\ | <INPUT TABINDEX=\ | <INPUT TABINDEX=\ | <INPUT TABINDEX=\ | 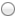 |
| **Q4g**- Le SIS permet d’être informé sur les soins et services reçus par un patient | <INPUT TABINDEX=\ | <INPUT TABINDEX=\ | <INPUT TABINDEX=\ | <INPUT TABINDEX=\ | <INPUT TABINDEX=\ | 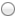 |
| **Q4h**- Le SIS permet une meilleure planification de vos activités cliniques | <INPUT TABINDEX=\ | <INPUT TABINDEX=\ | <INPUT TABINDEX=\ | <INPUT TABINDEX=\ | <INPUT TABINDEX=\ | 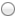 |
| **Q4i**- Le SIS permet d’éviter des investigations et examens inutiles | 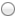 | 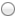 | 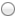 | 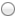 | 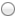 | 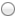 |
| **Q4j**- Le SIS facilite l’accès à l’information concernant les résultats d’un patient | 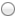 | 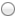 | 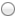 | 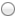 | 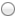 | 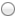 |

**Q5.** Quant à l’information fournie par le **Système National d’Information Sanitaire (SNIS)**, veuillez indiquer dans quelle mesure vous êtes en accord avec chacun des énoncés ci-dessous ?

|  | **Tout à fait en accord** | **Modérément en accord** | **Incertain (e)** | **Modérément en désaccord** | **Tout à fait en désaccord** | **Ne s’applique pas** |
| --- | --- | --- | --- | --- | --- | --- |
| **Q5a**- L'information fournie par le SNIS est complète | <INPUT TABINDEX=\ | <INPUT TABINDEX=\ | <INPUT TABINDEX=\ | <INPUT TABINDEX=\ | <INPUT TABINDEX=\ | 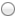 |
| **Q5b**- L'information fournie par le SNIS est exacte | <INPUT TABINDEX=\ | <INPUT TABINDEX=\ | <INPUT TABINDEX=\ | <INPUT TABINDEX=\ | <INPUT TABINDEX=\ | 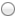 |
| **Q5c**- L'information par le SNIS est fournie rapidement | 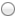 | 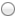 | 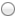 | 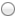 | 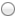 | 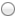 |
| **Q5d**- L’information du SNIS est disponible au moment où vous en avez besoin | 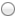 | 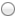 | 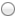 | 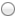 | 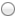 | 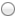 |
| **Q5e**- La présentation et la mise en page de l’information dans le SNIS sont adéquates | <INPUT TABINDEX=\ | <INPUT TABINDEX=\ | <INPUT TABINDEX=\ | <INPUT TABINDEX=\ | <INPUT TABINDEX=\ | 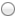 |

**Q6. Q6a**-Utilisez-vous actuellement un système d’information?


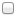
Oui


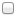
Non

**Q6.** Si oui, ce système est-il :

**Q6b**- compatible avec votre travail
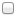
Oui


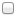
Non

**Q6c**- une surcharge à votre travail
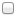
Oui


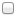
Non

**Q7.**Parmi ces fonctionnalité(s) du SIS, **lesquelles seraient utiles dans votre pratique**? Cochez tous les choix qui s’appliquent.


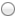
**Q7a**- Dossier patient électronique


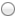
**Q7b**- Pilotage, statistiques et veille sanitaire / aide à la décision


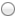
**Q7c**- Gestion des rendez vous


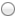
**Q7d**- Gestion des ressources

**Q7e**- Coordination, organisation et planification des soins

**Q7f**- Gestion administrative du patient

**Q7g**- Télémédecine (fonction déportée de l'hôpital)

Autre, préciser ___________________________

**Q8. Quant à la télémédecine adaptée au contexte du Gabon, veuillez indiquer dans quelle mesure vous êtes en accord avec chacun des énoncés ci-dessous ?**

|  | **Tout à fait en accord** | **Modérément en accord** | **Incertain (e)** | **Modérément en désaccord** | **Tout à fait en désaccord** | **Ne s’applique pas** |
| --- | --- | --- | --- | --- | --- | --- |
| **Q8a**- Elle réduit les évacuations sanitaires inutiles | <INPUT TABINDEX=\ | <INPUT TABINDEX=\ | <INPUT TABINDEX=\ | <INPUT TABINDEX=\ | <INPUT TABINDEX=\ |  |
| **Q8b**-Elle réduit les coûts liés à la référence pour le patient | <INPUT TABINDEX=\ | <INPUT TABINDEX=\ | <INPUT TABINDEX=\ | <INPUT TABINDEX=\ | <INPUT TABINDEX=\ |  |
| **Q8c**- Elle augmente l’attractivité des structures de santé |  |  |  |  |  |  |
| **Q8d**- Elle peut constituer une activité valorisante pour les centres experts |  |  |  |  |  |  |
| **Q8e**- Elle motive le personnel de santé | <INPUT TABINDEX=\ | <INPUT TABINDEX=\ | <INPUT TABINDEX=\ | <INPUT TABINDEX=\ | <INPUT TABINDEX=\ |  |

**Q9.** Indiquez votre profession

<INPUT TABINDEX=\ Médecin spécialiste

<INPUT TABINDEX=\ Médecin généraliste

Infirmier(ère)

Sage-femme

Gestionnaire

Autre profession de santé, préciser ____________________

**Q10**. Indiquez dans quel type d’organisation vous travaillez.

CHU

CHR

Centre médical

Centre de santé

Structure privée

Dispensaire

Autre, préciser ___________________________

**Q11.** Comment évalueriez-vous vos compétences en informatique en général ?

Aucune

Élémentaire

Moyenne

Avancée

Expert

**Q12.** À quel groupe d'âge appartenez-vous ?

<INPUT TABINDEX=\ Moins de 30 ans

<INPUT TABINDEX=\ 30 à 39 ans

<INPUT TABINDEX=\ 40 à 49 ans

<INPUT TABINDEX=\ 50 à 59 ans

<INPUT TABINDEX=\ 60 ans et plus

**Q13.** Quelle est votre ancienneté dans la profession que vous exercez actuellement ?

<INPUT TABINDEX=\ Moins de 5 ans

<INPUT TABINDEX=\ 5 à 9 ans

<INPUT TABINDEX=\10 ans et plus

**Q14.** Quel est votre genre ?

<INPUT TABINDEX=\ Féminin

<INPUT TABINDEX=\ Masculin

Merci de votre participation ! Vous pouvez indiquer vos commentaires ci-dessous.

....................................................................................................................................................................................................................................................................................................................................................................................................................................................................................................................................................................................................................................................................................................................................................................................................................................................................................................................................................................................................................................................................................................................................................................................................................................................
