# Supplementary material for: Implementation of a national electronic health information system in Gabon: a survey of healthcare providers’ perceptions
Source: BMC Med Inform Decis Mak. 2020 Aug 24;20:202. doi: 10.1186/s12911-020-01213-y (PMC7444076; doi:10.1186/s12911-020-01213-y)
Supplement: Supplementary file 3 — Additional file 3. Construct reliability of the model. [file 12911_2020_1213_MOESM3_ESM.docx]

Additional File 3. Construct reliability of the model

| Construct | Items | Label | CR | Factor Loadings |
| --- | --- | --- | --- | --- |
| Satisfaction |  |  | 0.90 |  |
|  | q1a | HIS performance |  | 0.81 |
|  | q1b | HIS ease of use |  | 0.82 |
|  | q1c | HIS quality of information |  | 0.88 |
|  | q1d | HIS support services quality |  | 0.8 |
| SupQual |  |  | 0.89 |  |
|  | q2a | The process of implementing HIS was adequate |  | 0.76 |
|  | q2b | The level of training in the use of HIS is adequate |  | 0.87 |
|  | q2c | The level of support offered for the use of HIS is adequate |  | 0.84 |
|  | q2d | Accessing the HIS at your workplace is easy |  | 0.79 |
| SQ |  |  | 0.92 |  |
|  | q3a | The response time of the HIS against a request is acceptable |  | 0.78 |
|  | q3b | The HIS is easy to use |  | 0.75 |
|  | q3c | The required effort to use the HIS is acceptable |  | 0.8 |
|  | q3d | The HIS ensures adequate privacy and security of patients' personal data |  | 0.84 |
|  | q3e | Access to the HIS is acceptable |  | 0.82 |
|  | q3f | The HIS has a reliable performance |  | 0.74 |
|  | q3g | The HIS allows you to enter all the information you want |  | 0.77 |
| Impact |  |  | 0.96 |  |
|  | q4a | The HIS increases your productivity |  | 0.71 |
|  | q4b | The HIS facilitates information exchange between different users of the system | | 0.77 |
|  | q4c | The HIS improves real time management of health care and services |  | 0.8 |
|  | q4d | The HIS improves patient information management in real time |  | 0.87 |
|  | q4e | The HIS provides easy access to the desired information |  | 0.86 |
|  | q4f | The HIS makes it possible to visualize the information related to a patient |  | 0.87 |
|  | q4g | The HIS allows to be informed about the care and services received by a patient | | 0.87 |
|  | q4h | The HIS allows for better planning of your clinical activities |  | 0.88 |
|  | q4i | The HIS avoids unnecessary investigations and examinations |  | 0.87 |
|  | q4j | The HIS facilitates access to information about a patient's results |  | 0.84 |
| IQ |  |  | 0.92 |  |
|  | q5a | The information provided by the HIS is complete |  | 0.76 |
|  | q5b | The information provided by the HIS is accurate |  | 0.82 |
|  | q5c | The HIS provides information quickly |  | 0.9 |
|  | q5d | Information is available in the HIS when you need it |  | 0.86 |
|  | q5e | The presentation and layout of information in the HIS are adequate |  | 0.86 |
